# On the Treatment of Uterine Inflammation

**Published:** 1861-07

**Authors:** E. J. Tilt


*On the Treatment of Uterine Inflammation by Nitrate of Silver and other Substitutive Agents.* By E. J. Tilt, M. D., M. R. C. P.

In using these agents, we only apply the general principles of therapeutics to the treatment of inflammatory affections of the womb and its associated organs. The utility of a solution of acetate of lead in inflammatory affections of the skin, implies similar utility in inflammatory affections of the vaginal mucous membrane; the utility of solutions of borax and of chlorate of potash in affections of the mouth, pointed to their trial as vaginal injections; the utility of sulphate of zinc and nitrate of silver in urethral strictures demonstrated by J. Hunter, Sir. E. Home, Lallemand, etc., suggested their employment in uterine catarrh; the sovereign utility of the solid nitrate of silver for the cure of cutaneous ulcerations, caused it to be tried in ulcerations of the womb. To whatever mucous membrane these agents are applied, they act in the same way—they substitute a therapeutical irritation, susceptible of being graduated to a morbid irritation, which might uncontrollably compromise the structures attacked—a temporary irritation to one that tends to become permanent.

In treating of injections, I have already enumerated several agents which act in this way, such as borax, chlorate of potash, acetate of lead, alum, sulphate of zinc, etc. They are generally given largely diluted, but if used in a solid or a highly concentrated state, the action of these agents would be analagous to that of two other important agents, nitrate of silver and tincture of iodine, which are called caustics by courtesy, but are no more caustics than tincture of cantharides. I wish it to be clearly understood that I hold these agents sufficient for the surgical treatment of uterine inflammatory diseases, in the large majority of cases, and so does Dr. H. Bennett, although it has been stated that we use strong caustics in ordinary cases of uterine disease. In a comparatively small number of instances, the structures of the womb have been too deeply modified by inflamation or by hyperthrophy, and are in so low a state of vitality, that the above named agents are insufficient to bring about the cure of disease. Then I have recourse to another class of substitutive agents, which are undoubted escharotics, for they cause a loss of substance proportionate to the amount of caustic used. Those caustics induce healthy acute inflammation in the tissues underlying the eschar, and, by judicious management of this healthy inflammatory action, the cure of chronic cases is often induced.

The caustics of which I shall treat are, the acid nitrate of mercury, potassa fusa cum calce, potassa caustica, and the actual cautery. At first sight it may seem strange to class together sulphate of zinc and potassa fusa cum calce, but one is justified in doing so, because the substitutive action which I ascribe to a solution of sulphate of zinc is pre-eminently shown in the results of potassa fusa cum calce, which often so raises the vital endowments of the uterine tissues as to promote rapidly healthy nutritive action in tissues which had been diseased for many years.

Such is my mode of practice, and I am glad to find that it accords, to a certain extent, with the practice of all whose opinion carries weight, and who, having in vain tried to cure uterine disease by nitrate of silver or milder measures, have recourse to one or the other of the strongest caustics. Dr. Fleetwood Churchill depends on nitric and muriatic acids, and on the acid nitrate of mercury, which is also preferred by Dr. E. Kennedy and by Dr. West; and although this distinguished pathologist considers ulceration of the cervix to be a condition of slight pathological importance, when he has to trace out a plan of treatment for his pupils, he has none other to propose than that already long ago carefully laid down by Dr. H. Bennett. The late Dr. Rigby, though adverse to the surgical treatment of uterine affections, admitted that there were certain cases of uterine ulceration requiring the use of potassa fusa or potassa fusa cum calce. Dr. H. Bennett prefers potassa fusa cum calce. This is not energetic enough for Professor Simpson, who uses potassa caustica; while the French strongly advocate a remedy older than Hippocrates, the actual cautery.

These comments upon the treatment of uterine inflammation will show that I am an eclectic, and that I use all the valuable agents which I have enumerated in certain cases which I shall specify. Again reminding the reader that I am not writing a treatise, I shall proceed to comment on the use of our pricipal substitutive agents.

*Tincture of Iodine.*—It is the ordinary tincture of the

Pharmacopœia which I mean, not the caustic tincture. I shall be brief on this agent, having already mentioned it as a revulsive, and having compared it with others then under discussion. Tincture of iodine seems to act as an astringent when slightly applied to the hypertrophied or inflamed surface of the neck of the womb, but as a vesicant, if several applications are made at one and the same time, and as a resolutive if re-applied every third or fourth day. It is much less useful than nitrate of silver as a topical application, but it suits better some idiosyncracies, and is well borne in diphtherical inflammation, when nitrate of silver should not be used. The fact that a solution of iodine can be injected into closed cavities and fistulous passages without severely inflaming them, marks it out as the best liquid to be injected into the body of the womb, in the very rare cases requiring such treatment; for it has less frequently given rise to the alarming symptoms of peritonitis, which have very often followed the intra-uterine injection of a solution of nitrate of silver. I use one drachm of the tincture to an ounce of distilled water, and inject it by means of an instrument similar to that devised by Mr. Coxeter for injecting fluids into the larynx.

*Nitrate of Silver.*—"The application of nitrate of silver is a means, under certain circumstances, of subduing external inflammation. Might it not, on this principle, be of service in the treatment of the internal phlegmasia?" Such was the question asked by Mr. Higginbottom in the preface of his admirable little work on "The Lunar Caustic," published in 1826. His question has been answered in the affirmative by a great many eminent practitioners, who have applied nitrate of silver for the cure of inflammatory affections of the mucous membrane of the eyes, ears, mouth, throat, urethra, the intestines, and the rectum. As regards the mucous membrane of the genital organs, Dr. Jewel, in 1830, strongly advocated its use; and I have no hesitation in saying that this agent is quite as useful in curing the varied inflammatory conditions of the genital organs as in curing those of the skin. It is often necessary to preface the use of nitrate of silver by linseed tea, poppyhead, or other cooling injections, in the same way that Mr. Higginbottom repeatedly inculcates the utility of cold poultices previous to applying nitrate of silver to the inflamed skin. If, after antiphlogistic treatment, the solid nitrate of silver increases too much habitual pains, or causes the ulcerated surface to bleed for two or three days afterwards, it is well to try a solution of from forty to sixty grains of nitrate of silver to an ounce of distilled water. In many cases the solution is sufficient to effect a cure; it gives less pain, but it may be necessary to repeat it every third or fourth day. Sometimes I use a solution of nitrate of silver containing one ounce of the salt to two or three ounces of distilled water, as an application to ulcerated surfaces. Chronic uterine catarrh, or inflamation of the mucous membrane lining the neck of the womb, which has been truly called an open gland pouring out mucus from ten thousand follicles, seems to me the most frequent of all uterine diseases. Without having the slightest abrasion, the mucous membrane lining the neck of the womb and its vaginal surface, may be of a dusky, livid hue, tender on being touched, and secreting pus. This condition may last for years, but it generally leads to more or less extensive denudation of the villi of the uterine mucous membrane, and gives an excoriated appearance to the neck of the womb. Such cases, with or without excoriation, can be cured by the nitrate of silver in solution, used every fourth or fifth day, with the occasional use of the solid nitrate. If the mucous membrane lining the cervix be principally affected, it is often so obstinate as to render the painting of it with the solution of little use. The solid nitrate must be freely employed, and when the cervical canal is unusually dilated, I sometimes leave about one-eighth of an inch in the canal; by which it will be clear that, so far as my experience goes, should the stick accidentally break in the cervical canal it need give no alarm. What cannot be removed will cause more pain, some loss of blood, and perhaps even a return of menstrua tion; but the patient may be repaid for greater suffering by a speedier cure. It has been stated by Nonat that this mode of treatment has caused stricture of the uterine canal, in his practice and in that of Richet. I have never met with this accident, and I think its occurrence is to be prevented by the occasional passage of the uterine sound for a few weeks after this severe application.

With regard to the treatment of the various forms of ulceration of the neck of the womb, I can add nothing to what has been so well laid down in Dr. H. Bennett's work. Mr. Higginbottom, whose statements with respect to the action of nitrate of silver, deserve the highest consideration, affirms that its action does not extend beyond three days after its application; and it is generally received that it is necessary to repeat the use of this agent so soon as the epithelial pellicle has fallen off, or every third or fourth day. In many instances this is the best way of insuring the most rapid recovery; but I do not recommend the too strict adherence to this precept, as it is often well to leave five, six, or seven days interval between the applications, or we might work as did Penelope, and retard the cure of the case. This, however, is a matter of surgical experience in each individual case.

Whether vaginitis occurs spontaneously or as the result of uterine catarrh, it is best cured by the injection of a solution of nitrate of silver. This is an excellent idea of Dr. Jewel, but if the solution be sufficiently strong to do good, it cannot be safely trusted to the patient. The patient being placed on her back, a small glass speculum should be introduced as far as possible, and an ordinary glass syringe, full of a solution of nitrate of silver, containing forty grains to the ounce, should be injected. The speculum should then be withdrawn to the vicinity of the vulva, and the fluid should be left in contact for three or five minutes, after which the speculum is to be withdrawn, and the fluid received in a small cup. Sometimes I apply a speculum of appropriate size, aud as I withdraw it I pretty freely touch the vagina with the solid nitrate of silver diluted by chloride of silver, as prepared by Mr. Squire. This is a modification of a plan recommended by Ricord.

I recommend these injections where there is evidence of inflammation of the womb, with excoriation of its cervix, in virgins in whom the integrity of the hymen prevents the introduction of a moderate-sized speculum. This plan should be first tried before forcibly dilating or incising the hymen, an operation which is very rarely required. I have frequently made these injections in many cases, and I do not once remember having traced menorrhagia to their administration. I mention this as it seems to have often occcurred in the practice of Dr. Fleetwood Churchill. So many serious accidents have followed the injection of the solution of nitrate of silver into the body of the womb, that I prefer using tincture of iodine in solution whenever intra-uterine injections may be required. In very rare cases of chronic internal metritis it may even be necessary to apply the solid nitrate of silver to the internal surface of the body of the womb, as well as to adopt other modes of treatment; for an account of which I refer the reader to my papers on the Treatment of Internal Metritis.*

In follicular inflammation of the labia, in eczema and prurigo pudendum, or proritus, both external and vaginal, a piece of cotton-wool should be soaked in the solution of nitrate of silver, and carefully rubbed for two or three minutes over the diseased portions of the skin and mucous membrane. I can speak with confidence of this plan, for I have lately cured several patients who had been suffering in this way for four,eight, and thirty years. When cases have lasted so long, the pudendal skin looks and feels like parchment. It was soi n the case of a lady in whom the disease had lasted thirty years, and I first rubbed in the solution every day, then every other day, then every fourth and fifth day, until the skin became soft and pliable, and the sleep was no longer disturbed by darts of pain flashing along the nerves. This

*The Lancet, 1553, vol. ii.

patient was cured in three months, and has had no relapse during the last year.

I trust I have said enough in praise of nitrate of silver; but in many forms of uterine inflammation, much more severe agents are required to restore the womb to a healthy state. This fact is admitted by so many authorities at home, in America, or in foreign countries, that I am surprised to find the contrary asserted by Dr. Meigs and Dr. Tyler Smith. After describing the evil effects of caustics in the treatment of uterine disease, the latter pathologist, in his work on "Leucorrhœa," (p. 203), gives, as his opinion, that "there is no good which can be effected by the more powerful caustics which cannot be accomplished by the nitrate of silver, or by other means. It is true, that by the prolonged application of the nitrate of silver loss of substance may be caused; but this is far less likely to occur with lunar caustic than with the more powerful escharotics. It is also true, that some practitioners apply the more violent caustics so lightly that they do not exceed the milder medical action of the solid nitrate of silver; but in such cases it would be quite as well to use the safer remedy where a caustic is required." And at p. 206, "In applying the nitrate of silver, the aim should be not to produce any slough or loss of substance." Thus it is clearly stated that the slight application of the strong caustics is tantamount to the full action of the nitrate of silver in like cases of uterine disease.

My experience, on the contrary, teaches me that nitrate of silver is no more a caustic than tincture of cantharides, as Mr. Higginbottom has long ago asserted. The distinction that Dr. Meigs draws between the antiphlogistic touches and the escharotic action of nitrate of silver, does not bear examination. Use it as you may, the nitrate of silver does not cauterize. Leave it in the neck of the womb, it will cause more pain, loss of blood, and subsequent discharge, but no destruction of tissue, unless coagulated mucus mixed up with epithelial scales and insoluble chlorides of silver can be called such. Even when applied to a fungous ulcer, the slight loss of substance is rather due to the friction of a hard body on a pulpy, than to the chemical combination of the neutral salt and the diseased tissues. A densely hypertrophied neck of the womb might be whitened with the solid nitrate of silver every fourth day until doomsday, without much reducing its bulk. Indeed I have seen such a plan of treatment injudiciously continued for a year or longer in a case of hysteralgia, the neck of the womb being healthy and of an average size, and the effects were rather astringent than caustic, condensing the tissues, narrowing the cervical canal, and rendering its diltation necessary and difficult. Thus, while nitrate of silver may be repeatedly applied without inducing a loss of substance, the slightest application of the potassa fusa to the neck of the womb produces an evident loss of substance; and, therefore, the two agents, however applied, produce totally different effects in similar cases. This is a question of surgical therapeutics which can be decided by any experienced surgeon. Writing on the treatment of stricture caused by gristly thickening of the urethral mucous membrane, Mr. Wade records his twenty-five years' experience of the comparative advantages of nitrate of silver and of potassa fusa, and he states: "I cannot let this opportunity pass without again calling attention to the fact, that the effects of the argentum nitratum and of the potassa fusa admit of no comparison, as they are totally dissimilar; that the former, when freely used, from its tendency to cause adhesive inflammation, has often been found to increase the urethral obstruction, whilst the remarkably solvent powers of the latter have no such tendency."*

The too free use of nitrate of silver to the inodular tissues of the urethra causes urethral stricture, as the too free use of the same agent to the cervical canal causes stricture of the neck of the womb, but without loss of substance. Indeed, if the whole range of diseases in which nitrate of silver is now used be passed in review, it will be found that it always acts by its dynamic, astringent, and antiphlogistic

*Wade, Stricture of the Urethra, 4th Edit., p. 117.

properties; whereas escharotics can only raise the standard of vitality of any given tissues by the previous destruction of their superposed surface. I maintain, on the contrary, that there is one good to be done with the more powerful caustics which cannot be accomplished by the nitrate of silver; that is, to shorten the treatment of many cases in which it is *at first* judicious to try it. Ulceration of the neck of the womb, on a hypertrophic basis, may doubtless be sometimes cured by the use of nitrate of silver, but the treatment might be indefinitely prolonged; whereas it can be very much shortened by one or two applications of the acid nitrate of mercury or of potassa fusa c. calce. When the inner cervix is chronically inflamed, nitrate of silver may enable us to effect a cure; but with that agent, however applied, cures are sometimes so tedious that it is well to resort to one or two applications of the acid nitrate of mercury or of potassa fusa c. calce. In fungous and varicose ulceration the nitrate of silver causes the surfaces to bleed profusely, and does more harm than good; whereas I find that in these cases the acid nitrate of mercury and the actual cautery stop the bleeding and promote a cure. I think it right to be sparing of caustics to the neck of the womb in pregnant patients; but I have seen cases similar to those described by Dr. Bennet in which it was necessary to stop an abundant purulent and bloody discharge from a large varicose ulcer, and I have done so with the acid nitrate of mercury after doing more harm than good with the nitrate of silver. So little are caustic agents and nitrate of silver interchangeable substances or therapeutical equivalents, that I find nitrate of silver in some cases to be positively poisonous, while potassa fusa c. calce conduces to recovery. In diphtheritic inflammation of the neck of the womb and of the vagina, nitrate of silver acts as a poison. In a case now under treatment there is a small patch of false membrane on the posterior lip of the os uteri, and around it are numerous ulcerations. Were I to touch them with nitrate of silver they would soon be covered with false membranes. Tincture of iodine would not produce this effect, neither would the potassa fusa c. calce; these, therefore, are the best means of curing this most tedious complaint, of which I have seen two instances, and Dr. Bennet three, and he would endorse what I affirm of such cases. Occasionally we meet with cases like two I am now attending, in which an extensive superficial excoriation of the neck of the womb bleeds profusely, and for the following two days, even when only touched with the solution of nitrate of silver, which likewise makes the sore more angry. In these cases I have nearly effected a cure by dressing the wound with tincture of iodine or the acid nitrate of mercury.

The profession is more and more convinced of the great utility of caustics in many diseases; were it otherwise, surgery would be deprived of a valuable remedy, and the obstetric art robbed of the only means of curing the most distressing cases of uterine inflammation. Patients would thus have to drag on from year to year their weary load of misery, with the only hope that the cessation of menstruation, by putting an end to the physiological liability of the womb, might also check its liability to inflammation.—*London Lancet for May.*

